# Allogeneic Antigen Composition for Preparing Universal Cancer Vaccines

**DOI:** 10.1155/2016/5031529

**Published:** 2016-10-03

**Authors:** Petr G. Lokhov, Elena E. Balashova

**Affiliations:** ^1^Department of Proteomic Research and Mass Spectrometry, Institute of Biomedical Chemistry, Moscow, Russia; ^2^BioBohemia Ltd., Moscow, Russia

## Abstract

Recently it was demonstrated that tumors induce specific changes to the surface of human endothelial cells thereby providing the basis for designing endothelial cell-based vaccines that directly target antigens expressed by the tumor endothelium. The present report extends these studies* in vitro* by investigating the efficacy of allogeneic antigens with regard to their ability to target immune responses against the tumor vasculature since alloantigens simplify vaccine development and implementation in clinical practice. We demonstrated that allogeneic SANTAVAC (Set of All Natural Target Antigens for Vaccination Against Cancer), which presents a specifically prepared composition of cell surface antigens from tumor-stimulated endothelial cells, allows targeting of the tumor vasculature with efficacy of 17, where efficacy represents the killing rate of target cells before normal cells are adversely affected, and efficacy of 60, where efficacy represents the fold decrease in the number of target cells and directly relates to tumor growth arrest. These data suggest that allogeneic SANTAVAC may be considered an antigenic composition that following administration in the presence of respective adjuvants may be clinically tested as a therapeutic or prophylactic universal cancer vaccine without adverse side effects to the normal vasculature.

## 1. Introduction

Vaccination using antigens expressed by endothelial cells lining the tumor vasculature represents the most attractive vaccination strategy because immunization using this approach may prevent the growth of any solid tumor [1]. Therefore, endothelial cells can be used as a source of antigens used in the development of a universal cancer vaccine (UCV). However, аutoimmune-mediated damage to microvessels, the primary targets of anticancer endothelial cell-based vaccination strategies, may lead to side effects, namely, autoimmune-mediated damage of microvessels in healthy tissues [2, 3]. Therefore, antigen compositions constituting a UCV that are distinct from antigens expressed by endothelial cells in normal tissues need to be designed to prevent the elicitation of undesired autoimmunity.

Recently we described the interactions between tumor-induced endothelial cell surface heterogeneity and endothelial cell escape from cell-mediated immune responses [4, 5]. These data sets suggested that an efficient autologous vaccine could be designed utilizing surface antigens expressed by cultured human microvascular endothelial cells (HMEC) if their tumor-induced cell surface profile and the profile of target HMEC were similar. In this scenario, the efficacy of the autologous vaccine would exceed 18 (i.e., 18 tumor endothelial cells will be destroyed before 1 endothelial cell in normal tissue is destroyed) [5]. Antigen compositions intended for vaccination were based on a specifically derived set of HMEC natural cell surface antigens distinguished by the abbreviation SANTAVAC (Set of All Natural Target Antigens for Vaccination Against Cancer) [6]. Although the design of these studies was mainly intended to describe an autologic type of SANTAVAC, it was found that alloantigen compositions also may be efficiently used for anticancer vaccination. In one case, the killing rate of target HMEC using allogeneic surface antigens related directly to the* in vitro* design of the allogeneic universal vaccine with efficacy of targeting equal to 4 [6]. This efficacy provides a therapeutic window where tumor HMEC could be killed before HMEC of normal tissues are adversely affected. Unfortunately, the efficacy of the allogeneic SANTAVAC in these studies was characterized by a limited number of experiments. To fill this gap, additional cytotoxicity assays (CTA) were performed in the present study to complement the UCV design based on the SANTAVAC. The possibility of excluding a patient's own biomaterial from the vaccine preparation simplifies the development activities and increases the value of the allogeneic SANTAVAC vaccines.

## 2. Materials and Methods

### 2.1. Cell Culture

Two abdominal subcutaneous adipose tissue biopsies were obtained from female patients (40–50 years old) undergoing open abdominal surgical procedures at the National Medico-Surgical Center (Moscow, Russia). The protocol was approved by the Research Ethics Committee and the patients provided written informed consent. The biopsy specimens were transported to the laboratory, and endothelial primary cultures were established using magnetic beads coated with anti-CD31 monoclonal antibody (Dynabeads CD31 Endothelial Cells, Invitrogen, Life Technologies, Carlsbad, CA, USA) as described previously [4]. Culture media (Medium MCDB 131 and Microvascular Growth Supplement, Gibco, Thermo Fisher Scientific, Waltham, MA, USA) supplemented with 10% FBS (fetal bovine serum) (PAA Laboratories, Dartmouth, MA, USA), 50 *μ*g/mL streptomycin, 50 U/mL penicillin, 2 mM glutamine (Gibco), and 12 U/mL heparin (Sigma-Aldrich, St. Louis, MO, USA) were changed every 2-3 days and after the first passage. Cells were grown to 65% confluence and used in future experiments. To obtain HMEC with tumor-induced phenotypes, cell cultures were incubated for 4 days in culture medium (MCDB 131, FBS, streptomycin-penicillin, glutamine, and heparin) supplemented with 5%, 15%, and 25% of tumor-conditioned medium. Cells were visualized using a Leiса DM5000B microscope (Leica Microsystems, Buffalo Grove, IL, USA).

Primary fibroblast cultures were established from an adult skin biopsy (45-year-old woman; donor provided written informed consent) as described by Rittié and Fisher [7]. Primary cultures were cultured in DMEM (Gibco) supplemented with 10% FBS, 5 *μ*g/mL streptomycin, 5 U/mL penicillin, and 2 mM glutamine at 5% CO_2_ at 37°C and third passage cells were used to obtain fibroblast-associated antigens (FAA).

### 2.2. Tumor-Conditioned Medium

Tumor-conditioned medium was collected from HepG2 (human hepatocellular carcinoma cells, ATCC, Manassas, VA, USA; cell lines have been authenticated by cell proteomic footprinting [8]) as described by Folkman et al. [9]. Media were conditioned for 48 h, collected, centrifuged for 10 min at 600 ×g, and filter-sterilized (0.2 *μ*m). Tumor-conditioned medium was then concentrated 10x using Centriplus Centrifugal Filter Devices YM-3 (Millipore, Merck KGaA, Darmstadt, Germany) and used in experiments.

In order to determine the optimal concentration of tumor-conditioned medium required to provide different tumor-induced stimuli to HMEC, the 10x tumor-conditioned medium was added to HMEC seeded in the wells of a 96-well plate at different concentrations (0, 10, 20, 30, 40, and 50% in MCDB 131 medium supplemented with streptomycin-penicillin, glutamine, and heparin). After 3 days in culture, cells were counted in wells using trypan blue staining to determine the concentration of tumor-conditioned medium that induced weak (stimuli just a little higher than in the control), moderate (half of the maximum), and strong (a little more than related to maximum) stimulation.

### 2.3. FACS Analysis

Endothelial cells were stained with phycoerythrin- (PE-) conjugated mouse anti-hVEGFR-2 IgG1 (clone 89106, R&D Systems, Minneapolis, MN, USA) or PE-conjugated mouse anti-human CD62E IgG1 (clone 68-5H11, BD Pharmingen, Becton Dickinson, San Jose, CA, USA). For isotype control, cells were stained with PE-conjugated mouse IgG1 (R&D Systems, clone 11711, or BD Pharmingen, clone MOPC-21, resp.). Flow cytometry was performed on a BD FACSCalibur flow cytometry system (Becton Dickinson) and the data analyzed using Cell Quest software (Becton Dickinson).

### 2.4. Preparation of SANTAVAC and FAA

HMEC or fibroblasts grown to 65% confluence were washed 5x with HBSS before being treated with 0.2 *μ*g/mL trypsin (15,000 U/mg, Promega, Madison, WI, USA) in HBSS. A 0.5 mL trypsin solution was added to each well of a 6-well plate, incubated for 20 min at 37°C in saturated humidity, then collected again, and centrifuged (600 ×g for 5 min). The resulting supernatant contained cell surface targets and was considered a solution of SANTAVAC or fibroblast-associated antigens (FAA), respectively.

### 2.5. Preparation of SANTAVAC-Loaded DC

Monocyte-derived dendritic cells (DC) were generated as described previously [10]. Briefly, fresh peripheral blood mononuclear cells (PBMCs) from healthy donors were isolated using Ficoll-Hypaque (PanEco, Moscow, Russia) gradient centrifugation and were then allowed to adhere to 12-well culture plates for 1 h. Nonadherent cells were collected and centrifuged, and cell pellets were mixed with autologous serum containing 10% DMSO and stored in liquid nitrogen. Cryopreserved, nonadherent PBMCs, which also are considered as peripheral blood lymphocytes, were later used as a source of effector cells (cytotoxic T lymphocytes, CTL) for cytotoxicity assays. The adherent cell fraction was cultured in RPMI-1640 (Gibco) supplemented with 10% FBS, streptomycin-penicillin, and glutamine in the presence of 0.075 *μ*g/mL granulocyte macrophage colony-stimulating factor (Neostim, 1.67 × 10^6^ ME, FDS FARMA, UK) and 1000 U/mL interleukin-4 (Sigma-Aldrich). After 6 d in culture, SANTAVAC (0.5 mL) or FAA (0.5 mL) were added to each well of a 12-well culture plate with immature DC (3 × 10^5^ cells/well in 1 mL of culture medium) and DC were matured with 1000 U/mL tumor necrosis factor-*α* (Sigma-Aldrich) for 48 h. Matured, SANTAVAC-loaded or FAA-loaded DC were then used to stimulate CTL.

### 2.6. Stimulation of CTL

SANTAVAC-loaded DC (3 × 10^5^ cells/well) in 12-well culture plates were combined with 6 × 10^6^ autologous nonadherent PBMCs (1 : 20) in 1 mL of RPMI-1640 medium (containing 10% FBS, streptomycin-penicillin, and glutamine) supplemented with 30 U/mL of clinical grade human interleukin-2 (Ronkoleukin, Biotech, St. Petersburg, Russia). The culture medium supplemented with interleukin-2 was replaced every third day. After incubation for nine days, nonadherent PBMCs containing stimulated CTL were washed by centrifugation and used as effector CTL in cytotoxicity assays.

### 2.7. Cytotoxicity Assays

HMEC (5 × 10^3^ cells/well) were seeded into 48-well plates, which yielded 3 × 10^4^ cells/well after 72 h. Effector CTL were then added to HMEC at an effector : target ratio of 20 : 1. On the third day, target HMEC were washed to remove CTL and attached HMEC were trypsinized and viability detected using trypan blue exclusion [11]. Cell counts were averaged over 3 measurements. The number of nonstimulated and tumor-stimulated HMEC in the presence or absence of effector CTL stimulated with FAA-loaded DC was used as controls. CTA data were used to calculate the* in vitro* efficacy of the allogeneic SANTAVAC formulation, namely, efficacy type I, denoted as “efficacy I” and calculated as a ratio of the number of* nonstimulated cells in control wells* (*i.e*., HMEC^0%^) to the number of tumor-stimulated cells in experimental wells, and efficacy type II, denoted as “efficacy II” and calculated as a ratio of the number* of tumor-stimulated cells in control wells* (*i.e*., HMEC^5%^, HMEC^15%^, or HMEC^25%^) to tumor-stimulated cells in experimental wells.

## 3. Results

### 3.1. Primary HMEC Cultures

**Figure 1:**
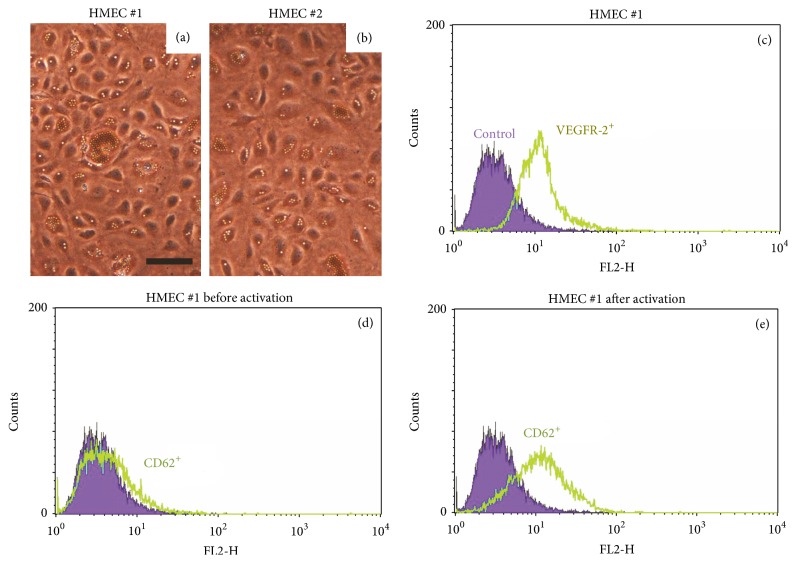
Primary HMEC cultures. A representative HMEC primary culture from donor 1 (a) and donor 2 (b). Images were obtained using a Leiса DM5000B microscope (scale bar, 50 *μ*m). Flow cytometric analysis of HMEC from donor 1 before activation (c, d) and after activation (e). Cells were stained with PE-conjugated monoclonal mouse anti-hVEGFR-2 or anti-human CD62E antibodies (labeled as “VEGFR-2^+^” or “CD62^+^”). For isotype control, cells were stained with PE-conjugated mouse IgG1 (labeled as “*control*”).

Anti-CD31 beads were used to isolate HMEC from a fat biopsy. Figures 1(a) and 1(b) show primary HMEC cultures isolated from fat biopsies obtained from donors 1 and 2, respectively. Adipose tissue-derived HMEC presented with typical cobblestone-like morphology. FACS analysis (Figure 1(c) for HMEC donor 1; data for donor 2 are not shown) revealed that the VEGFR-2 endothelial cell marker is associated with almost 90% of the cells during the first passage following isolation. No growth of contaminating fibroblasts or mesothelial cells was detected, demonstrating that primary HMEC cultures were successfully established. Additionally, FACS analysis of CD62 confirmed the endothelial nature of the primary cultures after activation using tumor-conditioned medium (Figures 1(d) and 1(e)).

### 3.2. Tumor-Induced Stimulation

**Figure 2:**
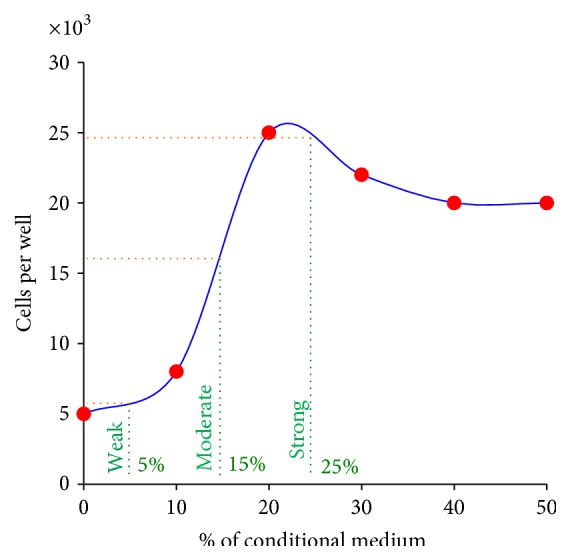
Dose determination of tumor-conditioned medium used to prepare HMEC with tumor-induced cell surface profiles. HMEC cultures were incubated with 0, 10, 20, 30, 40, or 50% tumor-conditioned medium. After 3 days in culture, cells were counted (red points) in wells using trypan blue exclusion. Cell numbers were approximated using a curve used to determine the concentrations of tumor-conditioned medium that elicited either* weak* (stimuli just a little higher than in the control),* moderate* (half of the maximum), or* strong* (maximum) stimulation of HMEC cultures (green lines). Green lines represent percentage selected for СTA.

The optimal concentration of tumor-conditioned medium required to provide HMEC stimulation was determined next. HMEC were cultured for 3 days in the presence of different concentrations of tumor-conditioned medium. Culture medium containing tumor-conditioned volumes of 5%, 15%, or 25% elicited weak (stimuli just a little higher than in the control), moderate (half of the maximum), or strong (a little more than related to maximum) levels of HMEC stimulation, respectively (Figure 2).

### 3.3. Cytotoxicity Assays

**Figure 3:**
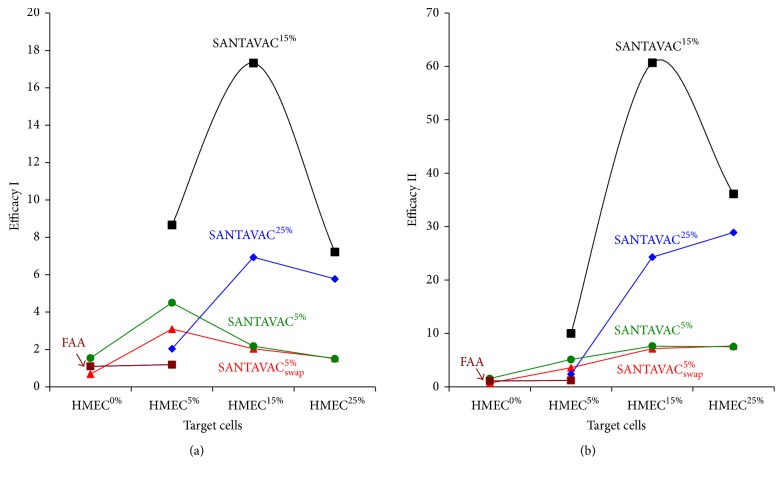
Efficacy I (a) and efficacy II (b) of target cell killing by SANTAVAC in CTA. Target HMEC were incubated in the presence of effector CTL at a 1 : 20 ratio. After 3 days, CTL were removed, target cells were carefully washed, and target cell viability was determined. Data is expressed as efficacy I (a) or efficacy II (b) of target cell killing by SANTAVAC. Efficacy I was calculated as a ratio of the number of* nonstimulated cells in control wells* (*i.e*., HMEC^0%^) to the number of tumor-stimulated cells in experimental wells. Efficacy II was calculated as a ratio of the number* of tumor-stimulated cells in control wells* (*i.e*., HMEC^5%^, HMEC^15%^, or HMEC^25%^) to the number of tumor-stimulated cells in experimental wells; that is, the percentage of tumor-conditioned medium in control wells was the same as in the experimental wells. Efficacy I allows* in vitro* estimation of the SANTAVAC efficacy by demonstrating how many endothelial cells in the tumor vasculature will be destroyed before 1 endothelial cell in normal tissue is destroyed (used to predict vaccine safety). Efficacy II allows* in vitro* estimation of the SANTAVAC efficacy by demonstrating the degree of HMEC proliferation suppression in the tumor vasculature and is used to establish the degree by which the vaccine can arrest tumor growth (predicted vaccine therapeutic effect). For efficacy calculation, the data representing the mean value of 3 independent measurements was used. “FAA” indicates the data related to the control (■) in CTA where fibroblast-associated antigens were used to simulate CTL. “*swap*” indicates CTA data where primary cell cultures used to generate antigens and primary cell cultures which were used as target cells were swapped (it was done to demonstrate the reproducibility of the CTA results at defined percentages of the tumor-conditioned medium used to stimulate HMEC). Percentage values indicated in the superscript correspond to the percentage of tumor-conditioned medium used to stimulate target HMEC or HMEC used to generate SANTAVAC for targeting immune response in CTA.

The immunologic properties of respective SANTAVAC compositions were evaluated by loading DC with corresponding SANTAVAC as a means of activating and stimulating human cytotoxic T lymphocytes (CTL) against target HMEC. CTL stimulated with fibroblast-associated antigen- (FAA-) loaded DC incubated in the presence of target HMEC were used as controls. On day 3, surviving target HMEC were identified using trypan blue exclusion. A subtle improvement in cytotoxicity was observed when CTL were stimulated with FAA-loaded DC. DC loaded with HMEC and stimulated with 15% or 25% tumor-conditioned medium elicited effective immune responses measured by high death rates of target HMEC (Figure 3). Notably, CTL stimulated with DC loaded with antigens from HMEC^15%^ (superscript represents the percentage of tumor-conditioned medium used to stimulate HMEC) was also most effective against HMEC^15%^ target cells (in this case almost all target cells were dead). The target HMEC^25%^ were most efficiently killed by CTL stimulated with DC loaded with antigens from HMEC^25%^. SANTAVAC efficacy I indicates that* in vitro* modeled vaccine safety was 17.3, achieved using antigens derived from HMEC^15%^ and HMEC^15%^ used as targets. SANTAVAC efficacy II indicates that the* in vitro* modeled capacity to arrest tumor growth was ~60, also achieved by SANTAVAC^15%^▸HMEC^15%^ (hereinafter [*SANTAVAC* ^*x%*^ *is SANTAVAC generated from HMEC* ^*x%*^ *for CTA*]▸[*HMEC* ^*y%*^ *used as target cells in same CTA*]).

## 4. Discussion

### 4.1. SANTAVAC: The Antigenic Composition of a Universal Endothelial Cell-Based Cancer Vaccine

Development of cell-based vaccines focuses on the elicitation of immune responses against target cells expressing native antigens [12, 13]. Cell surface targets are prioritized for vaccine design [14, 15] and are accessible to proteases whose byproducts could be isolated following* in vitro* proteolytic cleavage. Previously, it was shown that the “antigenic essence” of cells, which may be used in cell-based vaccines in contrast to whole cells, could be prepared by proteolytic cleavage of cell surface targets [16, 17]. The composition of this “antigenic essence,” which was established by the proteomic footprinting [8, 18], directly defined target cell killing rates in CTA that represent an* in vitro* anticancer vaccination model [4]. The “antigenic essence” prepared for vaccines designed to target the tumor vasculature gave rise to the name SANTAVAC [6]. SANTAVAC formulations can be mixed with different adjuvants and their immunogenicity and safety tested* in vivo* as UCV.

### 4.2. Allogeneic SANTAVAC

The present study expanded on the selection of alloantigens used in the preparation of SANTAVAC vaccines. The primary benefit of utilizing alloantigens as vaccine components is the possibility of excluding the patient's biomaterial from the vaccine preparation, thereby simplifying vaccine development, lowering the cost, and facilitating translation into clinical practice. Although autogenic SANTAVAC induced much higher target cell killing rates in CTA than alloantigens, alloantigens may also be highly efficient. Alloantigen compositions that induced low tumor killing rates also exhibited low killing rates of healthy tissues providing the required therapeutic window for their application. From the perspective of estimating vaccine efficacy (defined by target cell destruction in the absence of damage to healthy tissues), the immune response elicited by alloantigens was safer and may have broader applications in the medical field.

To prepare allogeneic SANTAVAC, the following previously discovered observations relating to endothelial cell heterogeneity were considered: (i) the tumor influence on HMEC was not specific to the tumor type and HMEC heterogeneity was a result of differences in strength of this influence; (ii) there was a linear dependence between target cell killing rates and the similarity of cell surface profiles of target cells and cells used to generate surface antigens for targeting the immune response; and (iii) the increase in tumor-induced changes at the HMEC surface led to decreased immunogenicity of HMEC surface antigens [4]. In addition, one particular observation from previous experiments suggested that the strongest changes to the HMEC surface were induced by HepG2 cells. This research was therefore designed to measure target cell killing rates in CTA where alloantigens were derived from HMEC stimulated to grow following stimulation by HepG2 that possessed a different signal strength (from weak to strong stimuli) [4]. It was therefore expected that the CTA experiments would reveal the maximum efficacy of allogeneic SANTAVAC* in vitro*.

Figure 2 showed how tumor stimuli strength was selected to provide HMEC with the diversity of tumor-induced surface profiles. Weak stimuli corresponded to the tumor-conditioned medium that induced an HMEC proliferation rate slightly higher compared to proliferation of control HMEC. Moderate stimuli corresponded to the percentage of tumor-conditioned medium which provided HMEC proliferation at half the maximum rate. Strong stimuli corresponded to the percentage of tumor-conditioned medium used which provided HMEC the stimuli to proliferate at a high rate.

### 4.3. Cytotoxicity Assays (CTA)

CTA revealed that HMEC with tumor-induced surface changes may be efficiently targeted by allogeneic SANTAVAC. This phenomenon was consistent with previously published data describing HMEC heterogeneity that established the foundation for the development of the SANTAVAC. Tumor cells induced unidirectional changes to the HMEC surface profiles resulting in a more similar antigen profile between target cell surface antigens and the surface antigen profile of cells used to generate antigens needed to target the immune response. As a consequence, the observed efficacy of SANTAVAC generated from HMEC^15%^ and HMEC^25%^ (i.e., SANTAVAC^15%^ and SANTAVAC^25%^, resp.) was sufficiently higher than the efficacy observed for control cells (*i.e.*, HMEC^0%^) and SANTAVAC^5%^. The high similarity between antigens present in SANTAVAC and the cell surface antigens expressed by target cells explains this observation.

The fact that target cell killing of SANTAVAC^15%^▸HMEC^15%^ was sufficiently higher than that of SANTAVAC^25%^▸HMEC^25%^ can be explained by one above-mentioned statement: that immunogenicity decreases with increasing tumor-induced changes to the HMEC antigen surface profile. Therefore, moderate tumor-induced changes to HMEC surface antigens would be preferable in the context of vaccine design resulting in efficacy I equal to 17.3 (safety) and efficacy II equal to 60 (capacity to arrest tumor growth). In this report two types of efficacy were described in relation to the allogeneic SANTAVAC vaccine. Efficacy I allowed for an* in vitro* estimation of the number of tumor vasculature endothelial cells that would be destroyed before one normal tissue endothelial cell would be destroyed. Efficacy II allowed for an* in vitro* estimation of the vaccine efficacy in the context of suppression of HMEC proliferation of the tumor vasculature and primarily is a reflection of the potential for the vaccine to arrest tumor growth; that is, it describes the vaccine's therapeutic effect.

It should be noted that stimuli of different strengths would be expected* in vivo* due to gradual diminishing growth stimuli in relation to increasing distance from the tumor cells. Therefore, it can be expected that HMEC with different target surface profiles, including profiles related to HMEC^15%^, will also be present in the tumor-associated vasculature.

## 5. Conclusion

Future studies in the field of vaccine development using allogeneic SANTAVAC are required; however,* in vitro* data presented in this report demonstrated that the allogeneic SANTAVAC was a perfect candidate for the development of a UCV with outstanding efficacy and safety. The SANTAVAC formulation described achieved efficacy equal to 17 and 60 in relation to* in vitro* prediction of vaccine safety and capacity to arrest tumor growth, respectively. Criteria critical to the development of such efficient allogeneic SANTAVAC are defined in this paper and may be used for preparing UCV for clinical trials.

## Abbreviations

HMEC: Human microvascular endothelial cells
DC: Dendritic cells
SANTAVAC: Set of All Natural Target Antigens for Vaccination Against Cancer
UCV: Universal cancer vaccine
CTA: Cytotoxicity assays
FAA: Fibroblast-associated antigens
CTL: Cytotoxic T lymphocytes.
